# Beyond 16S rRNA Community Profiling: Intra-Species Diversity in the Gut Microbiota

**DOI:** 10.3389/fmicb.2016.01475

**Published:** 2016-09-21

**Authors:** Kirsten M. Ellegaard, Philipp Engel

**Affiliations:** Department of Fundamental Microbiology, University of LausanneLausanne, Switzerland

**Keywords:** strain diversity, sub-species diversity, community analysis, gut microbiota evolution, metagenomics, honeybee

## Abstract

Interactions with microbes affect many aspects of animal biology, including immune system development, nutrition and health. In vertebrates, the gut microbiota is dominated by a small subset of phyla, but the species composition within these phyla is typically not conserved. Moreover, several recent studies have shown that bacterial species in the gut are composed of a multitude of strains, which frequently co-exist in their host, and may be host-specific. However, since the study of intra-species diversity is challenging, particularly in the setting of complex, host-associated microbial communities, our current understanding of the distribution, evolution and functional relevance of intra-species diversity in the gut is scarce. In order to unravel how genomic diversity translates into phenotypic diversity, community analyses going beyond 16S rRNA profiling, in combination with experimental approaches, are needed. Recently, the honeybee has emerged as a promising model for studying gut bacterial communities, particularly in terms of strain-level diversity. Unlike most other invertebrates, the honeybee gut is colonized by a remarkably consistent and specific core microbiota, which is dominated by only eight bacterial species. As for the vertebrate gut microbiota, these species are composed of highly diverse strains suggesting that similar evolutionary forces shape gut community structures in vertebrates and social insects. In this review, we outline current knowledge on the evolution and functional relevance of strain diversity within the gut microbiota, including recent insights gained from mammals and other animals such as the honeybee. We discuss methodological approaches and propose possible future avenues for studying strain diversity in complex bacterial communities.

## Introduction

All animals have evolved in a bacterial world, and interactions with microbes are known to affect many aspects of animal biology (Sekirov et al., 2010; McFall-Ngai et al., 2013). One of the major arenas for such interactions is the animal gut, which is typically colonized by a large number of diverse microbes (Ley et al., 2008b). Several important functions have by now been attributed to the gut microbiota, including host immune system development, nutritional supplementation, and pathogen colonization resistance (Round and Mazmanian, 2009; Sekirov et al., 2010; Lee and Hase, 2014).

The mammalian gut typically hosts hundreds of bacterial species, where the taxonomic composition varies both within and between host species (Ley et al., 2008a; Muegge et al., 2011; Delsuc et al., 2014; Sanders et al., 2015; Bik et al., 2016). Based on network analysis of gut taxonomic profiles in combination with host-specific traits, several parameters have been shown to play a role in shaping the composition of the mammalian gut microbiota, including diet, gut morphology and host habitat (Ley et al., 2008a; Muegge et al., 2011; Delsuc et al., 2014; Sanders et al., 2015; Bik et al., 2016). For example, herbivores and carnivores can be clearly distinguished by network analysis, consistent with the notion that the gut microbiota provides complementary diet-dependent metabolic functions to their hosts, e.g., the ability of herbivores to digest cellulose (Ley et al., 2008b; Douglas, 2014). However, examples of co-diversification of specific bacterial lineages with their mammalian hosts have also been found at shorter evolutionary time-scales (Falush et al., 2003; Moodley and Linz, 2009; Moeller et al., 2016), suggesting that host-adaptation also plays an important role in the evolution of the gut microbiota.

Despite variability in species composition, the mammalian gut microbiota is dominated by relatively few bacterial phyla, including Firmicutes, Bacteroidetes, Proteobacteria, Actinobacteria, and Verrucomicrobia (Ley et al., 2008a). Interestingly, thanks to an increasing number of 16S rRNA profiling studies, it is becoming clear that the same phyla recur in other metazoa, albeit with different relative abundances. For example, the Proteobacteria are abundant in both birds and fish (Sullam et al., 2012; Waite and Taylor, 2014), whereas they tend to have a lower abundance in mammals. Likewise, based on currently available data, both Proteobacteria and Firmicutes are common colonizers of the insect gut (Colman et al., 2012; Engel and Moran, 2013; Douglas, 2015). In contrast, free-living bacterial communities from other habitats, such as soil and water, have been shown to have a much broader representation of phyla (Ley et al., 2008b). Therefore, it appears that adaptation to life in the gut has occurred predominantly in a small number of bacterial phyla, which have diversified within the gut environment (Ley et al., 2006, 2008b).

**FIGURE 1:**
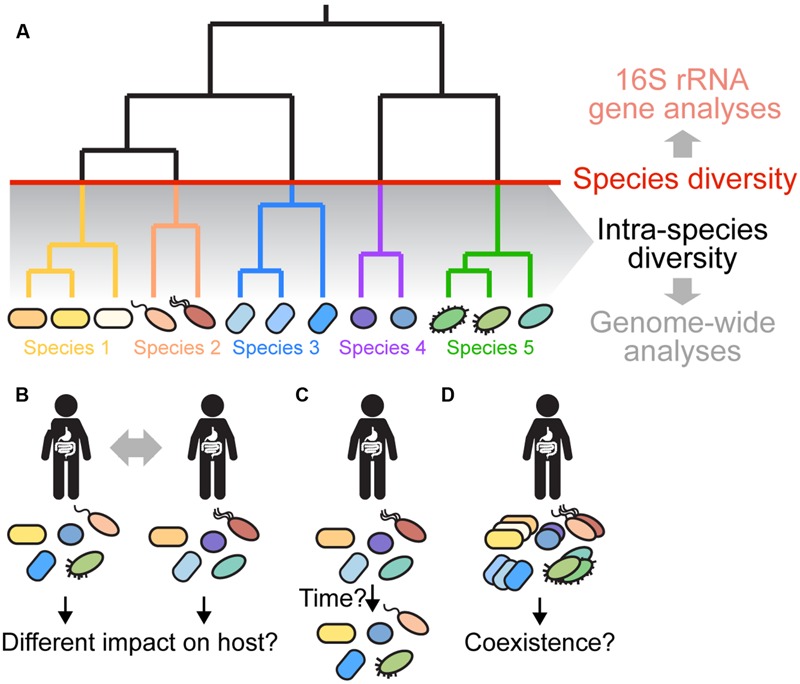
**Gut microbiota diversity beyond 16S rRNA gene profiling. (A)** Gut microbiota diversity is typically assessed at the species level (indicated by the red line) or higher based on amplicon sequencing of the 16S rRNA gene and clustering of sequences into OTUs using a 97% sequence identity cutoff. However, several recent papers have shown that most species have diversified into distinct strains and sub-lineages (shown in same colors) with marked variation in sequence and gene content (Faith et al., 2013; Schloissnig et al., 2013; Greenblum et al., 2015; Zhu et al., 2015). This intra-species diversity cannot be resolved using 16S rRNA-based analyses due to high sequence conservation of the 16S rRNA gene. Instead, genome-wide approaches are needed, because they allow for higher resolution and characterization of variation in the functional gene content among divergent strains. **(B–D)** Current questions about intra-species diversity in the gut microbiota are depicted. **(B)** What is the impact of intra-species diversity on gut microbiota function, and on the host? Are certain strains more beneficial than others? Does the combination of strains determine the impact on the host? **(C)** What are the dynamics and stability of intra-species diversity in the gut? Using low-error amplicon sequencing (LEA), it was recently shown that strains can persist in a given individual for years (Faith et al., 2013). Is this true for all gut bacterial species? How does the functional gene content change over time? Which factors contribute to the dynamics and long-term stability of intra-species diversity in the gut? **(D)** Which strains can coexist in a given individual? Have they adapted to different ecological niches or are they functionally redundant? What mechanisms are responsible for maintaining divergent strains in the gut? What are the dominant social interactions among divergent strains? Shape and color of bacterial cells depict the strain composition of a given individual. For simplicity of the representation, it is assumed that the species composition is the same in each individual.

Overall, 16S rRNA profiling studies have provided invaluable insights into the taxonomic composition of the gut microbiota, which in turn has facilitated the inference of broad evolutionary patterns. However, species diversity may only present the tip of the iceberg of microbial complexity in the gut. Recent studies applying methods that go beyond the typical 16S rRNA profiling approach have provided compelling evidence that the bacterial species of the gut microbiota are composed of a multitude of strains, which are likely to influence gut microbiota function (Faith et al., 2013; Schloissnig et al., 2013; Greenblum et al., 2015; Zhu et al., 2015) (**Figure 1**). Analysis of intra-species diversity is therefore needed to shed light on how horizontal gene transfer, competition and selection shape the evolution of gut-colonizing bacteria. In this review, we will outline current knowledge on intra-species diversity within the gut microbiota, discuss methodological approaches and propose possible future research avenues in this exciting new field.

## What is Intra-Species Diversity?

The bacterial species concept has been debated for decades (Wayne, 1988; Stackebrandt and Goebel, 1994; Sapp and Fox, 2013), and continues to be a contentious issue (Cohan, 2002; Caro-Quintero and Konstantinidis, 2012; Doolittle, 2012). Currently, there is no agreement as to what a bacterial species is, and it has even been proposed that the very concept is meaningless (Booth et al., 2016).

As in all organisms, bacterial evolution is shaped by genetic variation arising from mutations (Eyre-Walker and Keightley, 2007; Koskella and Vos, 2015). However, unlike multi-cellular eukaryotes, bacterial evolution is also strongly influenced by horizontal gene transfer (Ochman et al., 2000; Treangen and Rocha, 2011; Koskella and Vos, 2015). Although horizontal gene transfers happen more frequently between closely related bacteria, they also occur among distantly related species, and as such represent a major challenge for delineating bacterial species (Majewski, 2001; Fraser et al., 2007; Smillie et al., 2011). Indeed, bacterial strain diversity typically encompasses substantial gene content variation. To describe this bipartite nature of bacterial genomes, Tettelin et al. (2005) introduced the term “pan-genome” for the complete collection of genes that can be encountered in a bacterial species, while the genes shared by all members of a species was termed the “core genome.” In this early paper the comparative genome analysis revealed a core genome corresponding to approximately 80% of the total genome for each of the six sequenced strains of *Streptococcus agalactiae*. However, much higher levels of gene content variability have been found in later studies (reviewed by Land et al., 2015), perhaps in part as a consequence of the inflexible current species definitions.

Variability in gene content can be caused by either gene loss or horizontal gene transfer, where a large fraction of the variably present genes in a species can typically be attributed to phages and genes of unknown function (Ochman et al., 2000). Nevertheless, horizontally acquired (or lost) genes can also contribute to ecological adaptation, and they are likely to be major drivers of niche differentiation (and eventually speciation) among closely related bacteria (Smillie et al., 2011; Shapiro et al., 2012; Cordero and Polz, 2014; Biller et al., 2015; Bendall et al., 2016).

Variability in gene content among bacteria from the same species group was initially inferred from comparative genome analyses of cultured strains, typically isolated from samples collected at different time-points or from different locations (Rocap et al., 2003; Tettelin et al., 2005). However, during the last decade, studies of diversity directly from environmental samples have started to emerge, thereby providing novel insights into the occurrence of *co-existing* intra-species diversity in natural populations. Several studies have shown that natural communities harbor populations of bacteria, which form discrete clusters based on sequence analysis (Acinas et al., 2004; Venter et al., 2004; Fraser et al., 2007; Caro-Quintero and Konstantinidis, 2012). These studies are therefore consistent with the idea that discrete bacterial populations (akin to species) exist, but cannot be simply defined according to a universal sequence divergence cut-off. However, since a single environmental sample only provides a “snap-shot” of a population in time, the stability of the observed clusters cannot be known. Indeed, clusters would be expected to form in any population where lineages emerge and disappear (Fraser et al., 2007; Doolittle and Zhaxybayeva, 2009). This problem was recently addressed in a metagenomic study, where the authors followed bacterial populations in a freshwater lake over nine consecutive years (Bendall et al., 2016). Notably, evidence of both accumulation and purging of SNPs within populations was found, underscoring the importance of sampling at multiple time-points.

Still, as of yet, studies following natural bacterial communities over time-frames long enough to evaluate the coherence of bacterial populations are rare, and it is therefore not known whether similar patterns hold true in other bacterial populations or environments. For example, different populations might be expected to display different patterns of coherence depending on their propensity for recombination, which is known to differ by several orders of magnitude among species (Ochman et al., 2000; Fraser et al., 2007). Additionally, different habitats can be expected to support different levels of intra-species diversity and to be subject to distinct selection pressures.

In conclusion, while there is evidence from multiple studies to support that bacterial diversity is organized into discrete phenotypic and genetic clusters, there is no agreement as to how and whether such clusters correspond to bacterial species (Cohan, 2002). Meanwhile, however, scientists interested in grouping and analyzing the distribution of bacterial diversity from environmental samples seem to have arrived at a pragmatic solution; in the absence of a functional species concept, taxonomic profiling of bacterial communities is typically done by clustering 16S rRNA sequences into groups of 97% identity in the gene alignment (**Box 1**). These groups are subsequently referred to as “OTUs” (operational taxonomic units), thereby circumventing the need for the term “species.” Nevertheless, the 97% cut-off was originally proposed as a proxy for bacterial species, as it was found to correspond well to the 70% cut-off in DNA-DNA hybridization analysis (which was considered a molecular gold-standard for species delineation at the time; Stackebrandt and Goebel, 1994). Moreover, OTUs clustered at this level are generally analyzed as distinct functional units (akin to species), and several tools are available for estimating functional profiles from OTUs defined at this threshold (Langille et al., 2013; Asshauer et al., 2015). For simplicity, and due to the widespread application of the 97% sequence identity cut-off in community profiling studies, we will therefore refer to such groups as “species” or “species group” throughout this review, although we recognize the arbitrary nature of the classification. Furthermore, we will refer to all sequences falling within such groups as “strains.”

**BOX 1. 16S rRna Profiling and Intra-Species Diversity.**

Culture-independent taxonomic profiling of unknown bacterial communities is most commonly done by PCR amplification of the 16S rRNA gene, and has provided invaluable information on the diversity and complexity of the bacterial kingdom. However, for the purpose of studying intra-species diversity, the method suffers from the following limitations:

***PCR Artifacts***

Several primer-sets have been designed to target the 16S rRNA gene across bacterial phyla. However, while the goal is to target all bacteria, different primer-sets are known to generate different results, due to differences in annealing and amplification efficiency (Kuczynski et al., 2012). Additionally, PCR amplification can generate chimeric sequences and artificial SNPs due to polymerase errors. Therefore, an important initial step of any 16S rRNA pipeline is a stringent quality control, including trimming and filtering.

***Clustering and Analysis***

Theoretically, any unique short 16S rRNA sequence within a sample can be assumed to correspond to at least one distinct evolutionary lineage. However, in practice, the distinction between methodological artifacts (i.e., PCR and sequencing errors) and true diversity becomes non-trivial when the informative number of SNPs is small (Kunin et al., 2010). 16S rRNA sequences are therefore usually clustered into groups, which are referred to as OTUs. While any similarity threshold can in principle be applied, the most common cut-off is 97% identity in the 16S rRNA alignment, which is widely taken as a proxy for bacterial species (Stackebrandt and Goebel, 1994). Several clustering algorithms are available, and are known to generate different numbers of OTUs, particularly if higher similarity thresholds are applied (Schmidt et al., 2014). The common practice of clustering 16S rRNA sequences at 97% identity or higher therefore helps to reduce the inflation of OTUs caused by methodological artifacts, but at the expense of not resolving intra-species diversity.

## Intra-Species Diversity in the Gut Microbiota

Since standard 16S rRNA profiling provides little resolution for inferring intra-species diversity (**Box 1**), our current knowledge on the extent and distribution of intra-species diversity in the gut microbiota is scarce. However, pioneering studies have been conducted on the human gut microbiota, which have provided exciting new insights. Pilot studies have also been done on the honeybee, likewise unraveling the presence of extensive intra-species diversity. Thus, in the following, we will describe what is known about intra-species diversity in the gut microbiota, focusing primarily on these two model organisms.

### The Human Gut Microbiota

Culture-independent investigation of diversity in the human gut microbiota started with Sanger-sequencing of the 16S rRNA gene (Wilson and Blitchington, 1996; Suau et al., 1999), which provides relatively long sequence fragments. Therefore, although the highly conserved 16S rRNA gene provides low resolution power for distinguishing closely related bacterial strains (Stackebrandt and Goebel, 1994), the presence of intra-species diversity was already noted in early studies, as the number of unique long 16S rRNA sequences was found to be several orders of magnitude higher than the number of inferred species groups (Eckburg et al., 2005; Ley et al., 2006; Dethlefsen et al., 2007). However, due to the cost and labor associated with Sanger-sequencing, the overall sequencing depth was initially shallow, and it was evident that only a small fraction of the total diversity had been sequenced (Eckburg et al., 2005). With the advent of next-generation sequencing technologies, it has become possible to perform large surveys on hundreds of individuals, and to sample the gut microbiota deeply (Human Microbiome Project C, 2012). Unfortunately, the shift from Sanger-sequencing to next-generation sequencing technologies has also resulted in the generation of shorter read fragments, thereby reducing the information content available of intra-species diversity analysis even further (Stackebrandt and Goebel, 1994), while also increasing the impact of PCR/sequencing artifacts (Kunin et al., 2010).

Nevertheless, studies employing short-read shotgun metagenomics have shown that the functional profile of the gut microbiota can change significantly even when the taxonomic profile display minor changes, thereby providing indirect evidence for the functional relevance of intra-species diversity (Morgan et al., 2012; Qin et al., 2012). Indeed, if the functional profile changes in the absence of major changes in the species profile, replacement or addition of functionally distinct strains within pre-existing species groups can be presumed to have occurred. Considering the large accessory gene content typical of bacterial species (as defined in metagenomic samples based on the 97% cut-off in 16S rRNA identity), it is perhaps not entirely surprising that functional changes can occur in the absence of changes in the taxonomic profile.

In 2013, two large-scale studies were published, in which intra-species diversity was quantified in a more direct manner, providing a first estimate of the scope of intra-species diversity occurring in the human gut microbiota (Faith et al., 2013; Schloissnig et al., 2013). Firstly, these studies showed that the majority of species in the gut microbiota harbor multiple strains, also within individual hosts. Secondly, by following host individuals over time, both studies found that strains persisted within their hosts for years, indicating that strains can co-exist in the gut in a stable manner. Finally, strains were found to be largely host-specific, with similar strains being shared among closely related individuals.

These studies raised several important questions about the gut microbiota (**Figure 1**): what is the functional relevance of intra-species diversity in the gut? And how are host-specific profiles generated? On one hand, the host could potentially select strains with specific functional profiles. In fact, since the gut microbiota strain profile tends to be more similar among related individuals, it is even possible that strains could adapt to specific host backgrounds. On the other hand, host-specific strain profiles could also result from colonization bottlenecks early in life (Schloissnig et al., 2013), particularly if strains occupy similar niches in the gut.

Given the potentially large impact of early-life colonization events, several studies have by now been conducted on the infant gut microbiota. Overall, infants are known to have a more variable and less complex gut microbiota species composition than adults, with a higher abundance of bifidobacteria (Bäckhed et al., 2015). Nevertheless, based on comparisons between the gut microbiota of newborns and their mothers, it is clear that the maternal microbiota provides an important source of first-colonizers (Bäckhed et al., 2015). Whether strains acquired early in life also persist into adulthood or alternatively could be continuously exchanged among family members is not known. Interestingly, despite an overall low species complexity, the infant gut microbiota has also been shown to harbor extensive intra-species diversity (Sharon et al., 2013; Luo et al., 2015). Thus, Luo et al. (2015) found an average of 4.88 strains per subject in infants sampled during the first 3 years of life (as estimated based on SNP analysis within species groups).

In conclusion, several recent studies have reported on the presence of extensive intra-species diversity in the human gut microbiota. The functional relevance of this diversity is still unclear, but intra-species diversity has been associated with large differences in gene content, where variably present functions include transport, signaling and carbohydrate metabolism (Greenblum et al., 2015; Zhu et al., 2015).

With intra-species diversity being a common feature of the bacteria colonizing the gut, the molecular and experimental characterization of individual strains represents an enormous challenge. Moreover, most methods of strain characterization require culturing, which has widely been assumed to be an unrealistic prospect for the majority of gut bacteria. However, the “unculturability” of the gut microbiota has recently been challenged by two studies, where the authors in some cases were able to culture more than 90% of bacterial species present at more than 0.1% abundance (as estimated by 16S rRNA profiling; Browne et al., 2016; Lau et al., 2016). Thus, a wider range of tools for strain characterization of the gut microbiota have become available, which will be of great importance for elucidating the functional relevance of intra-species diversity in the human gut microbiota.

### The Honeybee Gut Microbiota

While the majority of studies on the gut microbiota have been conducted in mammalian model animals, invertebrates are becoming increasingly popular for functional studies, not least due to their experimental amenability. As for mammals, the invertebrate gut microbiota has been shown to play a role in nutrition and interactions with pathogens, indicating that these are general functional attributes of the gut microbiota of the metazoa (Dishaw et al., 2014; Douglas, 2015).

However, in contrast to most mammals, invertebrates typically host gut bacterial communities with much lower species complexity (Colman et al., 2012; Engel and Moran, 2013). With some notable exceptions, such as termites (Colman et al., 2012; Brune and Dietrich, 2015), the number of species in the invertebrate gut is generally less than 50 species per host (Colman et al., 2012). Thus, the sequencing of metagenomic samples derived from the invertebrate gut can provide a deeper coverage of all microbiota members, and thereby greatly facilitate the study of intra-species diversity.

For example, the honeybee (*Apis mellifera*) has been shown to harbor 8–10 bacterial species groups in the gut, which usually constitute more than 98% of the total bacterial community (Martinson et al., 2011; Ahn et al., 2012; Moran et al., 2012; Sabree et al., 2012; Corby-Harris et al., 2014). However, similarly to the human gut microbiota, the core gut microbiota of the honeybee has been shown to include an impressive amount of intra-species diversity (Engel et al., 2012, 2014; Ellegaard et al., 2015). Thus, in the first metagenomic study employing shotgun metagenomics on a single honeybee colony, *de novo* genome assembly resulted in multiple sequence variants of phylogenetic marker genes within species groups (Engel et al., 2012). Moreover, the length of the assembled contigs for each species was found to be several orders of magnitude higher than the expected genome sizes, indicating the presence of multiple co-existing strains within species groups. Consistently, sequencing of genomes from cultured strains of lactobacilli and bifidobacteria, isolated from a single apiary, revealed that around one third of the gene content in each sequenced strain was variably present within species groups (Ellegaard et al., 2015). Likewise, a surprisingly high extent of sequence divergence and gene content variation was identified by single-cell genome sequencing in two proteobacterial species of the honeybee gut microbiota, *Gilliamella apicola* and *Snodgrasella alvi* (Engel et al., 2014). Although most of the variable gene content is of unknown function, a strong enrichment of genes related to carbohydrate metabolism and transport was observed in all three studies, suggesting that the presence of intra-species diversity allow for higher metabolic flexibility in the community.

So far, comparative metagenomic studies have not been conducted on the honeybee gut microbiota. But, a gene phylogeny incorporating sequences from genome and metagenome data from two independent studies (Engel et al., 2012; Ellegaard et al., 2015) confirmed the presence of four distinct sub-lineages within the *Lactobacillus apis* species group at both sampling locations. These data therefore suggest that the intra-species diversity of the honeybee gut microbiota is structured around distinct evolutionary lineages, which may have different functional roles.

The mechanisms involved in generating and maintaining intra-species diversity in the honeybee gut microbiota are not known, and the distribution of intra-species diversity among individuals of a given honeybee colony has not yet been study in detail. However, based on full-length 16S rRNA gene sequences, Moran et al. (2012) could show that colony-specific strains seem to exist. Moreover, in another study from the same group, deep-sequencing of the protein-encoding gene *minD* revealed that most individual honeybees host more than one strain of the gut symbiont *Snodgrassella alvi* (Powell et al., 2016). Multiple functional and spatial niches are potentially available within honeybee colonies to support specialization and co-existence. For example, honeybees go through distinct life-stages and perform different tasks within the colony, which is also associated with distinct diets (Seeley, 1985). Thus, a recent study found significant changes in strain abundance for five out of 25 *Lactobacillus* strains between honeybees sampled at the age of 3 and 7 days old (based on differential abundance of unique 16S rRNA sequences; Anderson et al., 2016).

Whether the patterns of intra-species diversity observed in the honeybee also hold true for other invertebrate species is not yet known. However, based on diversity within 16S rRNA profiling data, the gut microbiota of the *Cephalotes varians* ant was also found to harbor extensive intra-species diversity, which in this case was found to distribute geographically (Hu et al., 2014). Likewise, early Sanger-sequencing of the 16S rRNA gene in termites revealed extensive intra-species diversity in the termite gut microbiota (Warnecke et al., 2007).

In conclusion, intra-species diversity has been described in the gut microbiota of both mammals and insects, and several studies have been published which indicate that intra-species diversity is likely to be functionally relevant. Future studies will show to what extent this is also the case for other animals and thus may be a general, recurrent pattern in gut microbiota evolution. In the following section, we will discuss possible mechanisms that may be involved in shaping and maintaining intra-species diversity in the gut microbiota.

## What Drives Strain Diversification, Coexistence, and Community Maintenance

A long-standing tenet in ecology, with roots dating back at least a century, hold that “complete competitors cannot coexist” (also known as “the competitive exclusion principle”; Hardin, 1960). Since strains of the same species are expected to have functionally overlapping gene repertoires, the co-existence of such lineages in natural communities is intriguing. Can closely related bacterial strains co-exist over long time-spans without out-competing each other, and if so, how?

### Micro-Niche Specialization

**FIGURE 2:**
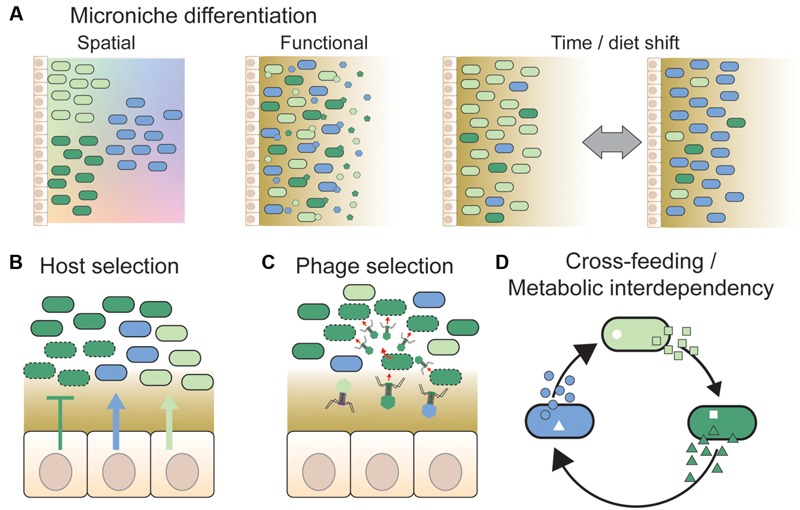
**Overview of different mechanisms hypothesized to facilitate strain co-existence. (A)** Divergent strains of a given species may be adapted to distinct micro-niches. These could be spatial niches, e.g., along nutrient or physicochemical gradients (shown by color gradients in the background) or according to variation in surface properties in different gut regions (not shown). Alternatively, strains may be adapted to functional niches, such as different types of dietary or host-derived nutrients. In this case, strains need not be spatially separated from each other. Temporal variation in environmental conditions, such as changing dietary habits or intake of drugs, may also facilitate co-existence by expanding the number of available niches and changing the fitness of strains continuously. This is also expected to generate temporal variation in strain abundance and diversity. **(B)** The host may select specific strains by the release of nutrients (arrows) or antimicrobial peptides (T bars) at the epithelial surface (Schluter and Foster, 2012), or by providing specific binding sites for bacteria (McLoughlin et al., 2016). Dashed outlines indicate killed or growth-arrested cells due to the action of antimicrobial peptides. **(C)** Phages have previously been proposed to play an important role in maintaining strain diversity in natural bacterial populations, via “kill-the-winner dynamics” (Rodriguez-Valera et al., 2009). Likewise, they may prevent massive expansions of strains in the gut by targeting abundant strains preferentially, thereby facilitating the maintenance of strain diversity (Barr et al., 2013). **(D)** Co-existence of bacterial strains may be facilitated by cross-feeding interactions, i.e., one strain produces a metabolite that is beneficial for another strain (Zelezniak et al., 2015). Such interactions may result in metabolic interdependencies, if the obtained metabolites are indispensable for bacterial growth and cannot be acquired or produced by other means (Morris et al., 2012). In conclusion, several mechanisms have been proposed to facilitate strain co-existence; notably, these mechanisms are not mutually exclusive, and may act together.

The simplest explanation for stable co-existence within the gut would arguably be that bacterial strains belonging to the same species group do in fact occupy distinct niches, despite being closely related. Indeed, several studies have shown that strains of the same species-group can be ecologically distinct within a shared habitat (Hunt et al., 2008; Gonzaga et al., 2012; Kashtan et al., 2014). Within the animal gut, such “micro-niche” specialization could be facilitated both by spatial heterogeneity and functional differentiation (**Figure 2A**).

The animal gut harbors a wide range of microbial habitats that vary in physiological parameters such as pH, oxygen and concentration of antimicrobial compounds (Donaldson et al., 2016). Thus, different bacterial families dominate the community of the small intestine and colon in humans (Donaldson et al., 2016), and the ileum and hindgut in honeybees (Martinson et al., 2012). However, marked local differences have also been demonstrated, i.e., between the outer mucus layer, inter-fold regions, colonic crypts and the luminal content in mice (Nava et al., 2011; Pedron et al., 2012; Li H. et al., 2015). Moreover, spatial structure in itself (i.e., patchy growth) is also expected to reduce the strength of interactions between populations and to dampen competition (Mitri and Foster, 2013; Coyte et al., 2015).

Functional differentiation, such as specialization toward different host- or diet-derived nutrients, may also facilitate co-existence between co-localized strains in the gut (**Figure 2A**). For example, strain diversity within *Bacteroides* spp. is associated with PULs (polysaccharide utilization loci; Xu et al., 2007; Zhu et al., 2015), which are used to breakdown distinct carbohydrates (Xu et al., 2007; Martens et al., 2014). Thus, although experimental studies on sugar utilization in *Bacteroides* spp. have so far been limited to cross-species comparisons (Rakoff-Nahoum et al., 2014, 2016), it is possible that such interactions also occur at the strain-level.

Finally, temporal variation in nutrient availability and composition may contribute to generating additional micro-niches within the gut (**Figure 2A**). Assuming that members of the gut microbiota can tolerate unfavorable conditions, at least for some time, a variable diet could potentially support a large number of specialized strains. For example, a large proportion of the human gut microbiota was recently shown to have spore-forming capabilities (Browne et al., 2016), which might ensure survival and resilience to perturbations. Moreover, temporal fluctuations in nutrient availability may also promote co-existence, by preventing any individual members of the gut microbiota from being at a competitive advantage over time-frames long enough to out-compete other community members (Gravel et al., 2011).

Since most of the gene content varying between bacterial strains is typically of unknown function, it is challenging to determine the extent of functional differentiation among strains of the same species group based on genomic data. Furthermore, it is unclear how much functional overlap can be tolerated under a scenario of stable co-existence. However, some studies suggest that subtle resource partitioning or functional differences may be sufficient for long-term co-existence (Tannock et al., 2012; Raghavan and Groisman, 2015).

### Host Selection

Unlike free-living bacterial communities, gut bacteria have to adapt to a biotic environment composed of a host, which may respond differentially to the presence of distinct bacteria. To what extent the host is able to control the composition of its gut microbiota is not known, but there is clear evidence for an on-going cross-talk between the host immune response and the gut microbiota (Rooks and Garrett, 2016; **Figure 2B**). For example, both mammals and insects secrete antimicrobial peptides in the gut, which affect bacteria differentially (Dishaw et al., 2014; Bunker et al., 2015; Donaldson et al., 2016; Planer et al., 2016).

From a host perspective, it is crucial to maintain a beneficial microbiota, and thereby exclude pathogenic strains. Moreover, a microbiota in which beneficial functions are present in diverse genomic backgrounds might increase productivity and insure robustness against disturbance (Yachi and Loreau, 1999). On the bacterial side, theory predicts that competition with other members of the gut microbiota will render costly extracellular functions susceptible to “cheaters” (Mitri and Foster, 2013). Therefore, host selection could potentially be important for the maintenance of beneficial functions of the gut microbiota.

Although the taxonomic profile of the gut microbiota varies both within and between host species, some studies have shown that gut bacteria colonize their native host better than other hosts (Kwong et al., 2014a; Seedorf et al., 2014). The mechanisms underlying this specificity are not clear, but it seems likely that bacterial outer-surface structures are involved. For example, intra-species diversity in adherence and aggregation factors correlates with host specificity in *L. reuteri* (Frese et al., 2011). Interestingly, while bacteria are expected to adapt their outer-surface structures to be able to colonize and attach to their host, it was recently proposed that the host might also exploit such structures to control the microbiota composition (McLoughlin et al., 2016). Specifically, since mammalian mucins are heavily glycosylated, and many bacteria have the ability to attach to glycans, the host could potentially promote and ensure the survival of beneficial bacteria via controlled glycan secretions (McLoughlin et al., 2016). Similarly, since several gut bacteria feed on epithelial-derived nutrients, it has also been proposed that the host may be able to promote the growth of specific groups of bacteria by providing selective nutrients (Schluter and Foster, 2012).

Based on these ideas, Donaldson et al. (2016) proposed that through “localized, immune-facilitated and adherence-dependent nutrient selection, the host maintains the stability of a diverse community of microbial symbionts.” However, whether such host selection can extend as far as to the maintenance of intra-species strain diversity in the gut remains speculative.

### Phage Selection

In an interesting twist, it has also been proposed that the maintenance of strain diversity at the epithelial gut surface is maintained by a third player, namely bacteriophages (Barr et al., 2013) (**Figure 2C**). Phages have previously been proposed to play an important role in maintaining strain diversity in natural bacterial populations, via “kill-the-winner” dynamics (Rodriguez-Valera et al., 2009; Koskella and Brockhurst, 2014; Thingstad et al., 2014; Takeuchi et al., 2015). According to this hypothesis, bacterial strains increasing in abundance are also more likely to be targeted by phages, thereby causing negative frequency-dependent selection. Although phages remain highly understudied, they do appear to be present in high abundance within the gut, just as in most other habitats (Ogilvie and Jones, 2015). Notably, in the study by Barr et al. (2013), a particularly high phage-to-bacteria ratio was found in the mucus layer of diverse animals (using epifluorescence microscopy). Moreover, bacterial attachment to mucus-producing cells was significantly reduced *in vitro*, when the mucus-producing cells were pre-treated with the mucus-adherent phage T4. Phage predation could therefore be of importance both for maintaining the mucosal barrier against invading pathogens, and for promoting and maintaining diversity within the gut microbiota.

Consistent with a phage predation model, Sharon et al. (2013) did indeed find patterns of co-variation in phage and strain abundance in the relatively simple gut microbiota of a premature infant. On the other hand, another study, likewise following strain abundance in the infant human gut microbiota over time, found that different species displayed different abundance dynamics, with some species maintaining a constant hierarchy of strain abundance over time, whereas other species displayed more variable strain abundance profiles (Luo et al., 2015). Furthermore, the strain abundance variation in one species group was accompanied by functional differences in sugar utilization genes, suggesting that dietary changes were involved, rather than phages.

As of yet, large-scale studies of co-variance in phage-strain abundance have not been conducted, since both quantification of strains and phages are technically challenging (Ogilvie and Jones, 2015). Notably, the presence of phages in the gut in itself need not result in “kill-the-winner” dynamics, since phages can also display a temperate life-cycle in which insertion into host genomes is preferred over host lysis (Reyes et al., 2010; Ogilvie and Jones, 2015).

### Inter-Dependence and Cross-Feeding Interactions

According to classic ecological principles, co-operation can be expected to be common among bacteria of the same genotype, whereas competition should prevail among different genotypes, i.e., strains of the same species-group (Mitri and Foster, 2013). Nevertheless, according to “the Black Queen Hypothesis,” recently proposed by (Morris et al., 2012), the evolution of co-operation between members in bacterial communities can be expected to occur if bacterial functions are “leaky,” resulting in the availability of “common goods.” Firstly, based on genome streamlining theory (Giovannoni et al., 2014), bacteria are expected to lose gene functions that are not strictly needed, due to a fitness advantage associated with avoiding the cost of maintaining the function. For example, as a proof of principle, it was shown that deletion of complementary metabolic genes for amino acid biosynthesis in two strains of *Escherichia coli* resulted in increased Darwinian fitness of the strains while grown in co-culture (Pande et al., 2014). Therefore, bacterial species may become dependent on other members of their community for providing specific functions. Moreover, if reciprocal losses occur, a network of inter-dependencies may evolve, preventing individual members from out-competing others (**Figure 2D**).

Since the presence of such dependencies is difficult to predict solely from genomic data, there is currently little evidence for their existence. However, while the impact of “black queen dynamics” on the evolution and maintenance of the gut microbiota is not known, cross-feeding interactions, whereby the waste-product of one bacterium becomes a nutrient source for another, are likely to be common in the gut (Fischbach and Sonnenburg, 2011; Zelezniak et al., 2015). Indeed, cross-feeding interactions have been demonstrated *in vitro* among members of the *Bacteroidetes* genus (Rakoff-Nahoum et al., 2014, 2016). Similarly, bacterial strains may benefit from each other’s presence without being strictly dependent on each other.

### Concluding Remarks

Several different mechanisms have been proposed to be involved in the maintenance of diversity within the gut microbiota (**Figures 2A–D**), but their relative importance for shaping and maintaining intra-species diversity is largely unknown. Is the gut simply an environment, which supports a high degree of micro-niche differentiation? Or do bacterial strains depend on or benefit from each other’s presence? And to what extent is the host involved in controlling and manipulating these highly complex communities? In order to answer these questions, we need to be able to quantify and follow the abundance of bacterial strains over time and in response to experimental manipulation, directly from bacterial communities. In the following sections, we will describe current methods employed to study intra-species diversity and dynamics directly from metagenomic samples.

## Culture-Independent Methods for Investigating Intra-Species Diversity

### PCR-Based Community Profiling

Taxonomic profiling of the gut microbiota is most commonly done based on PCR amplification of the 16S rRNA gene. However, for the study of intra-species diversity, the 16S rRNA profiling method provides limited resolution, and is susceptible to biases introduced by sequencing and PCR artifacts (Kunin et al., 2010; **Box 1**). To address this problem, Faith et al. (2013) developed a method termed “LEA-seq” (low-error amplicon sequencing). In this method, template sequences are tagged with barcodes in an initial linear PCR amplification step, followed by regular PCR amplification. After sequencing the final PCR products, the sequences are aligned in groups according to their barcodes, from which consensus-sequences can be derived and true variants can be inferred with much higher confidence. However, the read correction approach comes at the cost of a strongly decreased sampling depth, since each target has to be sequenced multiple times to obtain reliable consensus sequences.

Given the low information content of the 16S rRNA gene, primers targeting alternative genes have also been developed for studies on intra-species diversity. Although such primers can only target a subset of any community, they can provide a high level of resolution, and have been successfully applied to demonstrate the presence of intra-species diversity and co-diversification on shorter evolutionary time-scales in several recent papers (Lee et al., 2014; Caro-Quintero and Ochman, 2015; Moeller et al., 2016; Powell et al., 2016). However, regardless of the gene target, all PCR profiling methods are expected to induce skewed community profiles, due to differences in annealing and amplification efficiency of primers relative to their targets. Comparisons between samples can therefore only be done when the same primers and PCR conditions are applied. Moreover, in the case of the 16S rRNA gene, copy numbers differ widely between species, contributing further to skewed abundance profiles (Kembel et al., 2012).

Aside from PCR-associated artifacts, the targeting of single genes for diversity and evolutionary analyses is also affected by the variable rates of gene evolution in different bacterial species. For example, a comparison of the 16S rRNA identity and ANI (average nucleotide identity) scores between species showed that the core genome and the 16S rRNA gene evolve at different relative rates in different species (Kim et al., 2014). Therefore, even the 16S rRNA gene does not accurately reflect the evolution of bacterial communities. Finally, PCR-based profiling does not provide direct functional information pertaining to the diversity of a community.

### SNP-Based Analyses

Using shotgun metagenomics, sequencing data is obtained from complete genomes within a sample. Thus, although shotgun metagenomics has mostly been used to derive functional profiles of bacterial communities, this approach also has the potential to provide a high level of resolution for intra-species diversity analysis.

**FIGURE 3:**
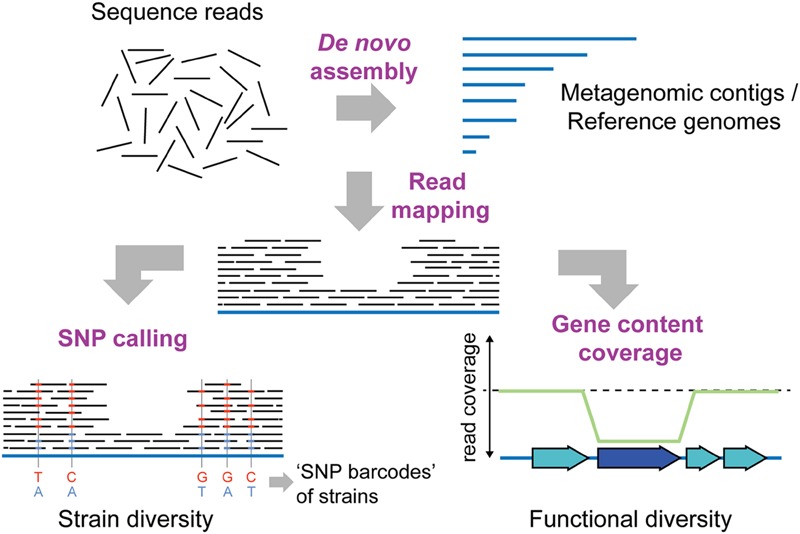
**Shotgun metagenomics to determine intra-species diversity in the gut microbiota.** Short-read sequencing technologies (100–150 bp), which provide high throughput and base-calling accuracy, are usually preferred for shotgun metagenomics. To infer intra-species diversity, sequence reads can be mapped to a database of reference sequences. Reference sequences can be genomes of previously sequenced cultured isolates or contigs obtained from *de novo* assembly of the metagenomic sequence reads themselves. Two distinct types of analyses are possible after read mapping: SNP (single nucleotide polymorphism) calling and gene content coverage analyses. SNP calling allows for determining the percentage of variable sites in reference sequences, and can provide insights into the extent of strain diversity for the different species in the community (in this case 5 sites, SNP variants indicated in blue and red colors). Based on the coverage of SNPs, strain-specific “bar-codes” may also be inferred (Luo et al., 2015). Thereby, the number of strains present in a sample can be estimated, and the abundance of strains can be followed over time in different samples. Alternatively, analysis of gene content coverage allows for determining gene sets and functions that vary in abundance in strains in the community. Genes not present in every strain in the community will have a lower read coverage than the remaining reference genome (dark blue gene). Similarly, genes present in multiple copies within the community will have a higher coverage than the rest of the reference genome (not shown).

Due to the targeting of complete genomes, shotgun metagenomics require a much higher sequencing effort than 16S rRNA profiling, in order to obtain a representative sample. Accurate high-throughput short-read sequencing technologies are therefore usually chosen. By mapping metagenomic reads, SNPs can be called on genes in reference genomes or on *de novo* assembled metagenomic contigs (**Figure 3**). Thereby, the extent of intra-species diversity can be estimated, and genes under positive or diversifying selection may be identified (Schloissnig et al., 2013; Bendall et al., 2016). For example, Schloissnig et al. (2013) were able to identify 10.3 million SNPs in 101 prevalent bacterial species, across 207 individuals, with bacterial species displaying highly variable levels of intra-species diversity. Moreover, by following 43 individuals over time, the authors could show that strain profiles were host-specific and persisted over a sampling period of 1 year.

While a global analysis of SNP diversity within metagenomic samples is of interest on its own, a higher goal would be to infer the presence of specific strains or lineages, and follow their abundance over time. A large number of genomes have by now been sequenced for many species, particularly from the human gut microbiota, which should facilitate this type of analysis. Unfortunately, the correct mapping of short reads among closely related strains is at best difficult. Tools have therefore recently been developed to improve the mapping accuracy in databases containing genomes from closely related bacteria, using statistical or probabilistic models to re-assign ambiguously mapped reads to their most likely origin genome (Hong et al., 2014; Ahn et al., 2015). In this manner, it is possible to follow the abundance and spread of known strains (or strains recently diverged from known strains), for example during a disease outbreak. Still, the efficiency of these tools is highly dependent on the availability of suitable reference genomes, since only strains with closely related sequenced genomes can be followed.

To reduce this dependency, Luo et al. (2015) recently published the tool “Constrains,” which attempts to quantify the most likely number of strains present in a sample. In this method, species with sufficiently high coverage for strain inference (set to 10x coverage) are initially identified using Metaphlan (Segata et al., 2012). Next, a custom database of representative marker genes is generated for each species, against which all reads are mapped. After mapping, the reference gene sequences are removed, and a table of coverage by base-position is created for all variable positions. Thus, only a single reference genome per species is necessary (provided that reads from divergent strains can still be confidently mapped to the genome). Since SNPs originating from the same strain are expected to have similar coverage within samples, model “barcodes” of SNPs with similar coverage can be generated and compared across samples, to estimate the most likely strain-specific SNP-barcodes (**Figure 3**). Moreover, when samples are available for multiple time-points, the abundance of strains (i.e., barcodes) can be followed over time.

In conclusion, SNP-based analysis of shotgun metagenomic data provides a powerful framework for quantifying intra-species diversity, selection and abundance dynamics. However, since all current SNP-based methods are based on core genes or marker genes, the functional profiles of quantified strains are not known, complicating the interpretation of abundance fluctuations.

### Gene-Content Analyses

Given the lack of functional information provided by PCR profiling and SNP calling methods, several recent methods have been developed to study intra-species gene content variation, based on shotgun metagenomics data.

**FIGURE 4:**
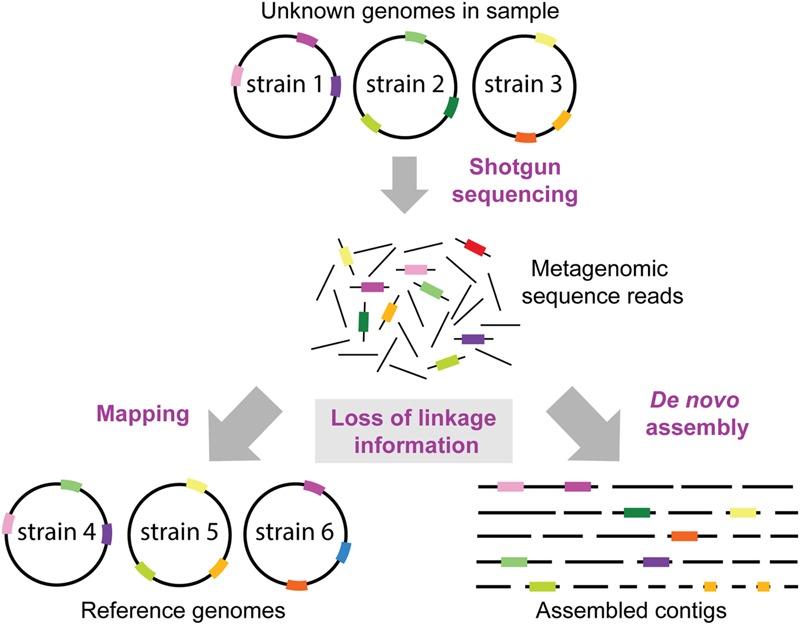
**Loss of linkage information in metagenomic datasets.** It is normally not known which strains and genomes are present in a metagenomic sample. Thus, the loss of linkage between genomic regions when generating short sequence reads presents a formidable challenge for reconstructing genomes of divergent strains, and to identify which genes or SNP variants (shown as colored bars on genome circles, reads and contigs) originated from which strain. Reference genomes may contain a different combination of genomic variants than the genomes in the sample resulting in reads mapping to the wrong reference genomes. Moreover, some regions (e.g., the dark green region) may not be encoded in the reference genomes in the database, and thus this information is lost when mapping the metagenomic reads. Likewise, *de novo* assemblies of short sequence reads will not result in complete reconstructions of the strains in the sample, but rather in multiple contigs which cannot be confidently linked to the same strain/genome.

In order to determine the gene content within a sample, the sequences can either by assembled *de novo* or mapped against a reference database (**Figure 3**). *De novo* assembly is desirable, since it allows for the discovery of novel genes or genomes in a sample. However, the assembly of short reads from complex bacterial communities is highly challenging. Firstly, the use of short reads is a general problem for genome assembly, since any repetitive sequences will hinder the assembly. This problem is exacerbated in samples from complex bacterial communities, where different species will have different optimal assembly parameters. Moreover, sequences from related strains may assemble into single or multiple contigs, depending on the conservation of the genomic region. Therefore, although several assemblers have by now been developed specifically for metagenomic data (Boisvert et al., 2012; Peng et al., 2012; Afiahayati and Sakakibara, 2015; Li D. et al., 2015; Nurk et al., 2016), metagenomic *de novo* assembly typically results in “a bag of genes” which are largely unlinked (**Figure 4**).

If a representative database of previously sequenced genomes is available, reference genome mapping can be a viable alternative. Relative to *de novo* assembly, read mapping represents a much less complex computational problem and several mapping tools are available that can efficiently handle even very large datasets (Hatem et al., 2013). However, due to the frequency of horizontal gene transfer in bacterial populations, reads mapping to the same genome in a reference database cannot be assumed to be linked within the sample. In fact, horizontal gene transfer is expected to be particularly frequent among closely related bacteria (Majewski, 2001). Thus, the genetic linkage within a metagenomic sample is lost in both *de novo* assembly and reference genome mapping (**Figure 4**).

Nevertheless, the presence/absence of genes in a metagenomic sample relative to a reference genome database can readily be determined, based on mapped read coverage (Zhu et al., 2015). Alternatively, gene content diversity within samples can be estimated by comparing mapped read coverage on genes to conserved single-copy genes, such as the universally conserved ribosome-related genes (Greenblum et al., 2015) (**Figure 3**). Similarly, when a suitable reference genome database is available, the pan genome can be estimated and genes with higher or lower coverage relative to the core genes identified (Scholz et al., 2016). Finally, the widely used tool “Metaphlan,” originally designed to characterize the species composition of metagenomic samples based on mapped read coverage of clade-specific marker genes (Segata et al., 2012), was recently updated to detect and track strains in addition to species (Truong et al., 2015).

In conclusion, all current methods for inferring intra-species gene content variation in metagenomic samples are based on mapped read coverage on genes, and therefore depend on the availability of a suitable reference database or a representative *de novo* metagenome assembly. In practice, it is challenging to evaluate the accuracy of these methods in real-life metagenomic samples; for example, mapped read coverage can depend on the location of genes within their host genomes, since growing bacterial populations will have a higher gene coverage if they are located near the origin of replication relative to the terminus (Korem et al., 2015). Finally, while gene content variation analysis does provide functional information at the community level, the identified variably present functions remain unlinked to their origin bacterial strains within the community (**Figure 4**, **Box 2**).

**BOX 2. Novel Sequencing Technologies For Metagenomics.**

Since a major obstacle for intra-species diversity analysis of metagenomic samples is the lack of linkage between SNPs/genes and their corresponding bacterial genomes (**Figure 4**), the development of sequencing technologies that can generate longer reads has the potential to greatly advance the field. Several novel technologies are currently under development, some of which have already become commercially available, with demonstrated improvements on applications such as *de novo* genome assembly (Chin et al., 2013; Loman et al., 2015). However, as of yet, short-read sequencing technologies are still preferred for metagenomic studies due to superior base-calling accuracy, high throughput and cost-efficiency.

Base-calling accuracy is fundamentally important for metagenomic studies, where multiple closely related bacterial strains are sequenced from the same sample. This requirement is currently not met by long-read sequencing technologies such as Single Molecule Real-Time (SMRT) sequencing (Chin et al., 2013) and Nanopore sequencing (Loman et al., 2015). In contrast, TruSeq synthetic long reads (TSLR) sequencing, recently introduced by Illumina, can generate long reads (8–10 kb) with very high base-calling accuracy (McCoy et al., 2014), making this technology interesting for metagenomic applications. Pilot metagenomic studies using TSLR reads have already yielded promising results, although taxonomic representation biases have also been noted (Sharon et al., 2015; Kuleshov et al., 2016).

Finally, metagenomic analysis targeting complete genomes also require very high throughput. Even with Illumina Hiseq sequencing, which is currently the most cost-effective and large-scale sequencing platform available, insufficient coverage is frequently a problem for strain-level analysis. For example, in a re-analysis of publicly available metagenomic data from the Human Microbiome Project, intra-species gene content variation was investigated for only 11 bacterial species, since all other species did not have sufficiently high coverage for reliable strain quantification (Zhu et al., 2015).

## Functional Relevance of Intra-Species Diversity – Future Avenues of Research

Based on several recent studies, it is now clear that intra-species diversity is omnipresent in the gut microbiota, also within host individuals (Faith et al., 2013; Schloissnig et al., 2013; Greenblum et al., 2015; Zhu et al., 2015; Kuleshov et al., 2016). However, little is known about how such diversity is generated or the mechanisms involved in obtaining, assembling and stabilizing diverse communities. Moreover, although several studies have shown that intra-species diversity also encompasses substantial gene content variation, the functional relevance of this diversity is not clear. A major problem in this context is the lack of accurate functional annotations, which is particularly common for genes that are variably present within species. Indeed, a major fraction of such genes typically cannot even be assigned a broad general function (Nayfach et al., 2015; Zhu et al., 2015; Oh et al., 2016). Therefore, in order to move the field forward from quantifying intra-species diversity to identifying causes and consequences of such diversity, we believe it will be crucial to link patterns of intra-species diversity to experimental and phenotypic data (Franzosa et al., 2015). To this end, experimentally amenable model systems with overall lower complexity of microbial diversity in the gut, such as the honeybee, can be a valuable addition to established vertebrate model species.

Although the honeybee is a relatively new model species for gut microbiota research, several tools have already been established (Kwong and Moran, 2016). Firstly, all members of the honeybee gut microbiota can now be cultured (e.g., Engel et al., 2013; Kwong and Moran, 2013; Olofsson et al., 2014; Kesnerova et al., 2016), and extensive frozen stock libraries are under development in several labs. Secondly, protocols for generating microbiota-free bees and re-colonizing bees have been established and applied successfully in a number of studies (Kwong et al., 2014a; Powell et al., 2014; Engel et al., 2015a). Thus, strain interactions can be queried both *in vitro* and *in vivo.* Thirdly, a large number of genomes isolated from the honeybee gut have by now been sequenced and analyzed, paving the way for future bioinformatic studies (Bottacini et al., 2012; Kwong et al., 2014a,b; Milani et al., 2014; Ellegaard et al., 2015; Engel et al., 2015b). Finally, in comparison to other established invertebrate models, such as *Drosophila*, the honeybee differs in being a social insect. Thus, the impact of social interactions on gut microbiota colonization and maintenance can be tested, either by restricting possibilities for social interactions experimentally (Powell et al., 2014) or by tracking interactions directly.

A combination of shotgun metagenomics and experimental data from animal models has the potential to provide novel insights into many of the currently outstanding questions concerning intra-species diversity in the gut microbiota (**Box 3**). In addition to providing distinct technical advantages, the use of diverse animal models will be of particular importance for understanding differences and commonalities of gut microbiota evolution across the metazoa. Likewise, a better understanding of fundamental mechanisms involved in maintaining and establishing diverse gut bacterial communities will be indispensable for establishing safe and effective probiotics to cure disease. For example, a recent study found that the success of colonization after fecal microbiota transplantation differed between hosts when using the same donor sample (Li et al., 2016). Moreover, bacterial strains from the donor sample were more successfully established in the new host when strains of the same species were already present (Li et al., 2016), highlighting once again the need to understand interactions at the strain level, also from an applied perspective. With the rapid development of sequencing technologies, computational tools and large-scale culture-based methods, exciting insights into the intricate relationships within natural bacterial communities will undoubtedly soon change and challenge the current views on our bacterial world.

**BOX 3. Key Questions Related To Intra-Species Diversity In The Gut Microbiota.**

• Evolution

◦ How do strains diversify in the gut?
◦ How do strains adapt to specific host environments?
◦ What is the impact of selection pressure exerted by the host versus other bacterial community members?

• Function

◦ Do specific strains contribute distinct complementary functions in the gut?
◦ Does the functional contribution of specific strains depend on host-specific factors or the presence of other community members?
◦ Does a high level of intra-species diversity benefit the host, i.e., by increased productivity or by providing robustness against disturbances?

• Community assembly and interactions

◦ How are communities with high levels of intra-species diversity established and maintained?
◦ Do strains of the same species group occupy distinct niches in the gut?
◦ Do strains of the same species group compete or collaborate?
◦ Which mechanisms facilitate stable co-existence within the gut?

## Author Contributions

All authors listed, have made substantial, direct and intellectual contribution to the work, and approved it for publication.

## Conflict of Interest Statement

The authors declare that the research was conducted in the absence of any commercial or financial relationships that could be construed as a potential conflict of interest.
